# Meals on Wheels? A Decade of Megafaunal Visual and Acoustic Observations from Offshore Oil & Gas Rigs and Platforms in the North and Irish Seas

**DOI:** 10.1371/journal.pone.0153320

**Published:** 2016-04-14

**Authors:** Victoria Louise Georgia Todd, Jane Clare Warley, Ian Boyer Todd

**Affiliations:** 1 Ocean Science Consulting, Ocean House, 4 Brewery Lane, Belhaven, Dunbar, East Lothian, United Kingdom; 2 Institute of Sound and Vibration Research, University of Southampton, Southampton, Highfield, Hampshire, United Kingdom; Virginia Commonwealth University, UNITED STATES

## Abstract

A decade of visual and acoustic detections of marine megafauna around offshore Oil & Gas (O&G) installations in the North and Irish Seas are presented. Marine megafauna activity was monitored visually and acoustically by Joint Nature Conservation Committee (JNCC) qualified and experienced Marine Mammal Observers (MMO) and Passive Acoustic Monitoring (PAM) Operators respectively, with real-time towed PAM in combination with industry standard software, PAMGuard. Monitoring was performed during routine O&G industrial operations for underwater noise mitigation purposes, and to ensure adherence to regulatory guidelines. Incidental sightings by off-effort MMOs and installation crew were also reported. Visual and acoustic monitoring spanned 55 non-consecutive days between 2004 and 2014. A total of 47 marine mammal sightings were recorded by MMOs on dedicated watch, and 10 incidental sightings of marine megafauna were reported over 10 years. Species included: harbour porpoise (*Phocoena phocoena*), Atlantic white-sided dolphin (*Lagenorhynchus acutus*), white beaked dolphin (*Lagenorhynchus albirostris*), common dolphin (*Delphinus delphis*), minke whale (*Balaenoptera acutorostrata*), common seal (*Phoca vitulina*), grey seal (*Halichoerus grypus*) and, basking shark (*Cetorhinus maximus*). Passive Acoustic Monitoring was conducted on two occasions in 2014; 160 PAM hours over 12 days recorded a total of 308 individual clicks identified as harbour porpoises. These appear to be the first such acoustic detections obtained from a North Sea drilling rig whilst using a typically configured hydrophone array designed for towing in combination with real-time PAMGuard software. This study provides evidence that marine megafauna are present around mobile and stationary offshore O&G installations during routine operational activities. On this basis, Environmental Impact Assessments (EIAs) for decommissioning O&G platforms should be carried-out on a case-by-case basis, and must include provisions for hitherto overlooked marine megafauna.

## Introduction

Despite a recent fall in oil prices, the global demand for Oil and Gas (O&G) exploration and production continues to increase. During 2014 in the UK alone, £14.8 billion was spent exploring for (and developing) O&G reserves [1], with a current 776 platforms operating in the North Sea today. Over the next twenty years, however, the industry faces a growing number of redundant O&G installations being taken out of service. In the UK North Sea sector, estimations of cumulative taxpayer expenditure for decommissioning range from £30–75+ billion over the next 30 years, depending on sources consulted (e.g. Oil & Gas UK, Oil & Gas Authority, Decom North Sea, etc.). Decommissioning is thus at the forefront of industry and Governmental agendas, but complete removal is a highly complex activity that has currently unknown and quantified Health, Safety, Environmental (HSE), financial, political, and social implications.

Leaving O&G structures in situ as artificial reefs is a potential alternative, and is known as a Rigs-to-Reefs (RTR) scheme. The concept began as early as 1975, when in Malaysia, the storm-damaged Baram-8 platform was toppled and made into an artificial reef [2]. Since then, RTR schemes have been implemented successfully in Brunei [3] and the United States’ Gulf of México [4]. RTR was legislated as an option in the State of California (and is gaining scientific credence from a variety of different research approaches), but opposition has prevented its implementation [5–8].

Despite growing evidence that North Sea O&G installations aggregate and produce marine life [9–23], current Oslo and Paris Convention (OSPAR) legislation prevents any part of the structure being left in the marine environment at the end of an installation’s operational lifetime, except for the derogation of some gravity-based installations (such as Shell’s Brent Field). OSPAR’s ‘clean seabed’ rules are up for review in 2018, and further data are required urgently to determine impacts of installations, and assessments of potential RTR schemes, on specific species and the ecosystem as a whole. On this basis, several projects have been initiated such as INfluence of man-made Structures In The Ecosystem (INSITE), Living North Seas Initiative (LiNSI), and Scientific and Environmental ROV Partnership using Existing iNdustrial Technology (SERPENT).

Traditionally, baseline studies around artificial reefs have focused on developing and testing models with time and spatial dimensions, which predict ecosystem changes caused by artificial habitat manipulation. This approach is difficult practicably when dealing with transient jack-up rigs that remain in one location only temporarily. A localised increase in abundance of potential megafaunal prey species (and 500 m fishing exclusion zones) make rigs and platforms potential foraging locations for top level predators protected from incidental catch in fishing nets. Moreover, jack-up rigs peregrinate from location to location, representing potential ‘mobile’ foraging locations. Peer-reviewed research on marine mammals around anthropogenic offshore structures, however, is limited to a handful of studies [24–26].

Access to rigs and platforms is limited due to strict security restrictions, so data collection within the 500 m exclusion zone are rare. Many marine mammals, such as harbour porpoises, are difficult to detect visually, so surveys beyond exclusion zones are likely to miss individuals foraging close to the legs. Similarly, the high frequency nature of harbour porpoise echolocation clicks (peak frequency 128–131 kHz [27–29]) means that individuals foraging close to installation’s legs are undetectable from acoustic monitoring surveys outside this zone. Consequently, studies from installations themselves are required to gain a better understanding of the importance of these structures to harbour porpoises and other marine megafauna.

Dedicated marine megafauna studies around offshore installations are also rare; however, as part of drilling and Vertical Seismic Profiling (VSP) permit applications, mitigation in the form of MMO and PAM observations are often stipulated, particularly in Special Areas of Conservation (SAC). While observations are constrained mostly to periods of a few hours to days covering the O&G industrial noise period of concern (e.g. conductor driving), over time, they can develop into substantial datasets. Any resultant raw data are sometimes submitted to the regulator, but more often confined to confidential reports, representing a valuable, but overlooked resource documenting the occurrence of marine megafauna species around offshore installations. This paper presents visual and acoustic evidence of marine megafauna present around mobile and stationary offshore installations in the North and Irish Seas.

## Materials and Methods

### Study locations and timings

**Fig 1:**
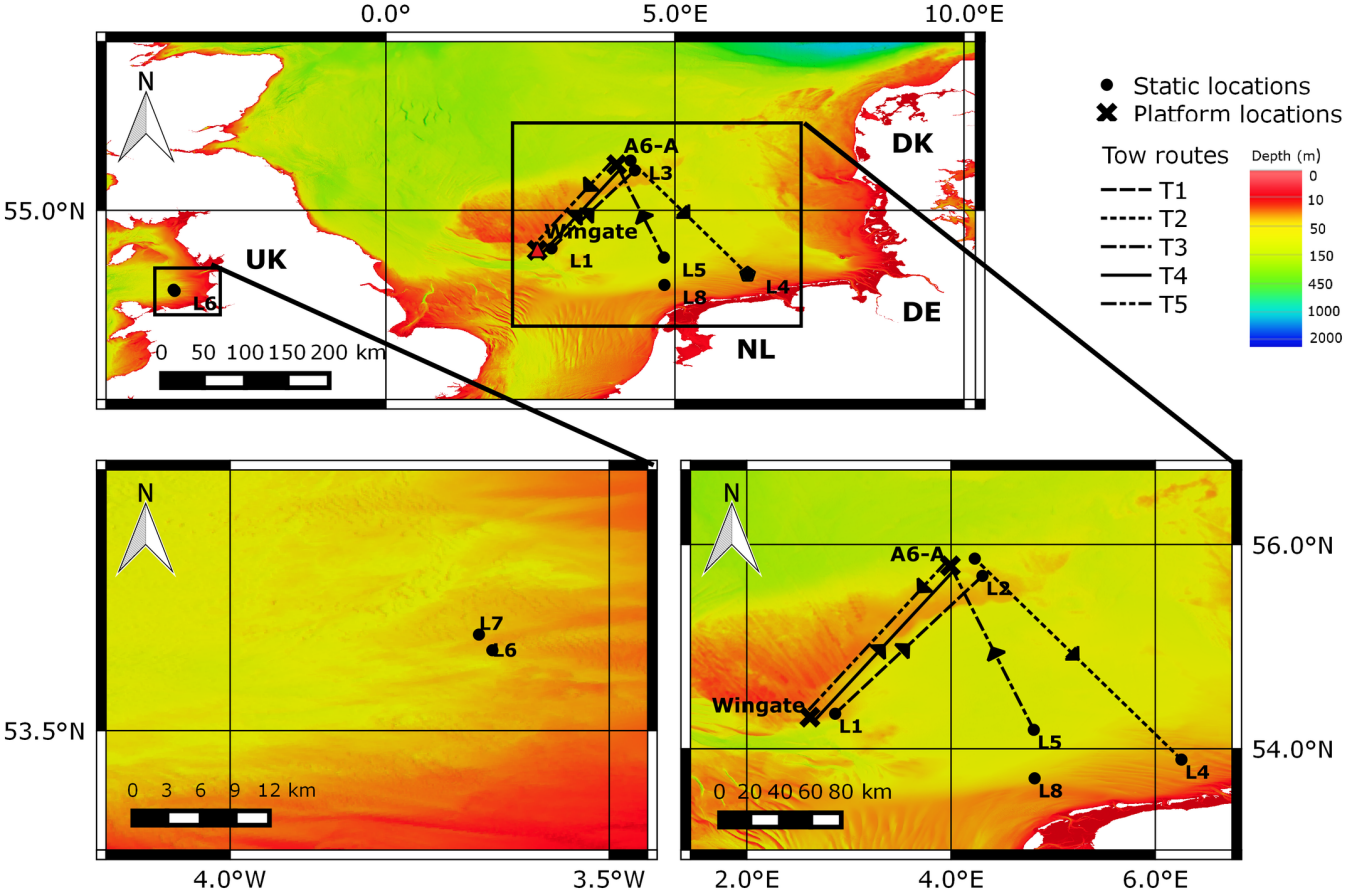
Location of production platforms, static monitoring locations, and tow routes. Bottom left = Irish Sea; bottom right = North Sea; UK = United Kingdom; NL = Netherlands; DE = Germany; DK = Denmark; L = location; T1 = tow one from L1 to L2 in 2004; T2 = tow two from L3 to L4 in 2009/2010; T3 = tow three from L5 to A6-A in 2012; T4 = tow four from Wingate to A6-A in 2014; T5 = tow five from A6-A to Wingate in 2014. Bathymetric metadata and Digital Terrain Model data products have been derived from the EMODnet Bathymetry portal [30], as per S1 File, and chart produced in QGIS [31].

Between 2004 and 2014, visual and acoustic monitoring for marine mammals was undertaken from two offshore O&G production platforms and four jack-up exploration drilling rigs, whilst stationary and under tow, in the North and Irish Seas. Operations were conducted in Germany, The United Kingdom, The Netherlands and Denmark. Permission for work carried out in Germany was granted by the German Federal Environment agency (UBA), in the UK by Department of Energy and Climate Change (DECC), in The Netherlands by the Ministry of Infrastructure and the Environment and in Denmark by the Danish Environmental Protection Agency. Production platforms had been in situ for 4–16 years, but jack-up drilling rigs were towed between locations to drill new wells, either independently of, or whilst attached to, a platform. Details of platforms and locations are displayed in Table 1 and Fig 1. The A6-A and Location 4 (L4) are located in the Dogger Bank Special Area of Conservation (SAC) and Borkum Riffgrund SAC respectively.

**Table 1:** Details of production platforms and static monitoring locations.

Name	Location	Country	Coordinates
A6-A	North Sea	DE	55°47.48’ N 03°59.66’ E
Wingate	North Sea	UK	54°18.56’ N 02°37.10’ E
Location 1	North Sea	NL	54°20.59’ N 02°51.96’ E
Location 2	North Sea	DE	55°41.57’ N 04°18.50’ E
Location 3	North Sea	DK	55°51.74’ N 04°13.99’ E
Location 4	North Sea	DE	53°53.60’ N 06°15.47’ E
Location 5	North Sea	NL	54°11.11’ N 04°48.70’ E
Location 6	Irish Sea	UK	53°36.61’ N 03°39.41’ W
Location 7	Irish Sea	UK	53°37.60’ N 03°40.30’ W
Location 8	North Sea	NL	53°42.61’ N 04°49.17’ E

DE = Germany, UK = United Kingdom, NL = Netherlands, and DK = Denmark. Coordinates in World Geodetic System 1984 (WGS ‘84).

Approximately 237 hours of visual monitoring was conducted throughout five Tows (T1–T5: 152 hours), and during six static (S1–S6: 85 hours) monitoring periods, and approximately 160 hours of acoustic monitoring was conducted over two tows (T4–T5); reported incidental sightings were also recorded. ‘Tows’ refers to when visual and/or acoustic monitoring was conducted from a jack-up drilling rig that was in transit (using tugs) from one platform or location to another, and ‘static’ is used to describe monitoring conducted from stationary production platforms, solitary jack-up drilling rigs, or production platform-jack-up drilling rig complexes. Incidental sightings are those recorded by off-effort MMOs and PAM Operators or by installation crew. Details of tow routes are displayed in Table 2 and Fig 1, and static monitoring periods in Table 3. Tow routes are plotted as straight lines between start and end coordinates, since MMOs are not obliged to track rig position en route.

**Table 2:** Details of visual and acoustic surveys undertaken whilst under tow.

Tow	Rig	Dates	Duration (days)	Visual Effort (hh:mm)	Acoustic Effort (hh:mm)	Tow start location	Tow end location
T1	NK	29/10/04–04/11/04	7	50:47	—	L1	L2
T2	NGS	26/12/09–03/01/10	9	54:25	—	L3	L4
T3	NGS	15/01/12–17/01/12	3	18:55	—	L5	A6-A
T4	NGS	10/01/14–15/01/14	6	14:34	63:11	Wingate	A6-A
T5	NGS	27/06/14–02/07/14	6	13:33	97:02	A6-A	Wingate

Visual = Marine Mammal Observer (MMO); Acoustic = Passive Acoustic Monitoring with towed hydrophone array & PAMGuard software [32]; NK = Noble Kolskaya; NGS = Noble George Sauvageau.

**Table 3:** Details of visual surveys undertaken from static locations.

Static	Rig	Dates	Duration (days)	Effort (hh:mm)	Monitoring method	Location
S1	NK	17/12/04–21/12/04	5	10:44	Visual	L2
S2	A6-A	04/08/05, 12/08/05 & 28/01/06	3	05:45	Visual	A6-A
S3	NGS	17/02/10–21/02/10	5	36:37	Visual	L4
S4	E 80	25/05/13–26/05/13	2	06:25	Visual	L6
S5	E 80	30/07/13	1	05:24	Visual	L7
S6	E 121	29/09/14–02/10/14	4	20:22	Visual	L8

Visual = Marine Mammal Observer (MMO); NK = Noble Kolskaya; NGS = Noble George Sauvageau; E80 = ENSCO 80; E121 = ENSCO 121

### Installation descriptions

A6-A production platform specifications are given in [24]; Wingate platform specifications are available at [33]. Four jack-up drilling rigs were utilised during the 10-year period. Noble Kolskaya (NK) is described in [24], and Noble George Sauvageau (NGS; now the Paragon HZ-1) at [34]. Specifications of both ENSCO 80 and ENSCO 121 jack-ups can be viewed at [35].

### Weather conditions

Empirical onsite weather data, including wind speed (m/s) and direction (°), sea state (Beaufort), swell height (m), sun glare and precipitation were collected by MMOs and PAM Operators whilst on effort. Wind speed and direction were sourced from installation weather stations, and remaining variables estimated by eye, using standard Beaufort windscale descriptive keys (e.g. [36]) by experienced MMOs and PAM Operators.

Additional forecast weather variables were collected for all tows, and for S1, S2 and S3. Forecast data for T1, S1 and S2 was sourced from [37], and from [38] for T2 –T5 and S3. Forecasts for significant wave height (m), swell height (m), wind speed (m/s), wind direction (°), air temperature (°c), and sea temperature (°c) were recorded four times per day, at 00:00, 06:00, 12:00, and 18:00.

On some occasions, empirical weather data were also sourced from proximate North Sea buoys, which were chosen based on nearest location to platforms and static monitoring locations. Details of weather buoys are shown in Table 4.

**Table 4:** Details of North Sea weather buoys.

Buoy name	Location	Distance from installation/location (m)	Monitoring period	Data sourced	Source
Borkum Noord	053° 45.15’ N 006° 37.06’ E	28,820 (L4)	T2; S3	Significant wave height sea temperature	[39]
Station 62145	053° 06.18’ N 002° 48.00’ E	3,000 (A6-A)	T3	Wind speed; significant wave height	[40]
South North Sea	054° 19.00' N 002° 55.99’ E	200 (Wingate)	T4	Wind speed; wind direction; air temperature	[41]
South East North Sea	055° 25.00' N 003° 49.00' E	430 (A6-A)	T5	Wind speed; wind direction; air temperature	[41]

Coordinates in World Geodetic System 1984 (WGS ‘84)

Forecast weather data were ground-truthed to buoy data, to assess accuracy. Data were tested for normality and parametric tests undertaken where applicable (e.g. Pearson’s product moment correlations). If transformation failed to normalise data, non-parametric statistics (e.g. Spearman’s Rank Order Correlations) were applied where appropriate.

Significant wave height, swell height, wind speed, and Beaufort sea state were chosen as the most useful parameters to indicate extreme or clement weather conditions, and thus those that have most impact on ability to monitor marine mammals visually. Given the sporadic nature of MMO and PAM data collection, there is limited scope for statistical analysis, but weather conditions are displayed graphically, and compared qualitatively to sea state.

### Operational activity

Where monitoring was carried out for specific operations, e.g. Vertical Seismic Profiling (VSP) or conductor driving, operational activity data were collected by MMOs and PAM Operators according to mitigation guidelines, and recorded in Excel^™^ databases. Variables included: sound source start and end times, details of soft-starts, sound source testing, and times of reduced power output. Sound source characteristics such as frequency and intensity were also collected.

When possible, more specific details about rig and platform activities were included from official logs maintained by installation crews. Daily logs are kept routinely on rigs, so data are complete for required periods, but operational logs concerning potential ‘noise-source’ activities are rare aboard platforms, as they merely produce oil and/or gas the majority of the time; consequently, operational logs from platforms are often missing or incomplete

Data were collated into databases, and divided into categories, described in Table 5. Operational activity at the time of a sighting is displayed graphically.

**Table 5:** Jack-up drilling rig and production platform operational activity categories.

Activity	Description
**Drilling Rig**	
Completion & well test	Prepare a well for production, including establishing a flow line for hydrocarbons
Drilling	Mechanical process in which a well-bore is drilled into the seabed
On tow	Rig on tow from one platform/location to another
Preparing for tow	Preparation of rig move
Waiting on Weather	Operations on hold due to weather; likely conducting general maintenance activities
Wirelining	Process of lowering/lifting equipment and measurement devices into and out of the reservoir for well intervention, reservoir evaluation, and pipe recovery
Conductor driving	Process by which piles are hammered into the seabed
**Production platform**	
Production	One, two, or three (etc.) well production

### Marine Mammal Observation

Operations and weather permitting (Beaufort sea state 1–4; good visibility >5 km), two Joint Nature Conservation Committee (JNCC) qualified and experienced MMOs conducted visual observations throughout daylight hours using standard JNCC techniques [42, 43]. Observer location varied between monitoring periods, but MMOs were positioned generally to ensure a 360° field-of-view around the sound source (e.g. conductor driving, drilling, etc.). During tows, MMOs scanned the horizon from 0° to ±90° of the installations’ heading. Observer platform height ranged from 13–60 m.

Scans were conducted using hand-held 7x50 binoculars in combination with the naked eye. Range estimations for sightings were calculated using either a graduated pole (T1 and S1) or reticle binoculars (T2–T5 and S2–S6), along with standard trigonometric techniques [44].

Data were entered into standardised JNCC recording forms. Watch start and end time, Global Positioning Satellite (GPS) location, water depth, barge tow speed, and empirical weather data were recorded on effort forms. Details of all sightings were entered into sightings forms, and where possible, photographs taken for identification purposes down to species level. Over the decade, installation crews’ incidental sightings of marine megafauna were reported to MMOs inconsistently.

The purpose of industrial MMO and PAM data is to identify species presence for mitigation purposes only [45]; therefore, seasonal comparative statistical analysis has not been undertaken, and sightings are not compared quantitatively to operations. Locations of all sightings are plotted using Geographical Information Systems [31].

### Passive Acoustic Monitoring

Towed, real-time PAM was deployed from the NGS in 2014 only. A 200 m potted hydrophone array was used to monitor for harbour porpoises around installations, see Table 6 for specifications.

**Table 6:** Towed hydrophone array specifications.

Array type	No. of elements	Spacing (m)	Flat frequency response (3dB points; kHz)	Hydrophone sensitivity (dB re 1V/μPa)
Seiche Measurements Ltd. potted towed array	4	0.25 (H1 & H2)1.2 (H2 & H3) 1.2 (H3 & H4)	2–200 (H1, H2, H3)0.075–30 (H4)	-166 (H1, H2, H3) -157 (H4)

H = hydrophone.

Two trained, qualified, and experienced PAM Operators carried out acoustic monitoring during T4 and T5. On both occasions, the hydrophone array was located on a reel stand on the port aft deck of the NGS. The cable was deployed directly over the side. Depth of deployment varied from 3–29 m and <1–29 m for T4 and T5 respectively. Depth fluctuations were unavoidable, due to wake turbulence associated with a non-hydrodynamic barge shape; array location was constrained to minimise interference with ongoing rig activities.

High frequency sounds were sampled using a National Instruments 6251 sound card, set to monitor two channels (Hydrophones 1 and 2). A sampling rate of 500 kHz was used. Porpoise echolocation data were processed using the click detector module in PAMGuard [32], with settings optimised for harbour porpoises (pre-filter: high-pass Butterworth filter set to 40 kHz; trigger filter: Butterworth band-pass filter set to 125–150 kHz, trigger threshold 10 dB). Low-mid frequency sounds were sampled using an RME Fireface 800 soundcard, set to monitor two channels (Hydrophones 3 and 4). A sampling rate of 48 kHz was set; vocalisations were viewed real-time on a scrolling spectrogram.

PAMGuard was configured to record continuously at all times; data were recorded as.wav files. All PAMGUard configuration settings, and module outputs were also stored in an Access database, and as binary data. All recordings in the 10–200 kHz band were viewed retrospectively in PAMGuard viewer by two experienced PAM Operators independently, and all clicks identified as harbour porpoises by PAMGuard were examined to ensure correct classification. Clicks were defined as harbour porpoises if they satisfied the following criteria: 1) significant energy around 130 kHz band [27–29]; 2) polycyclic waveform resembles that of published data for harbour porpoises [27, 28, 46]; and 3) narrow band with a -3dB bandwidth of 6–26 kHz [27–29]. Acoustic detection location is given as GPS coordinates of hydrophone array location; no attempt was made to estimate range or bearing to detections, as per left right ambiguity restrictions of in-line hydrophone arrays [45].

A click event was defined as two or more clicks separated by <60 s as per [47]. Number of minutes in which porpoises were detected (Porpoise Positive Minutes; PPM) were also calculated and locations of detections on tow were plotted in GIS [31]. Inter-Click-Intervals (ICI) were assessed to determine the presence of inferred feeding behaviour [24], and as per [24] a minimum ICI (MICI) of <10 ms, was used as a proxy indicator of possible feeding buzzes.

## Results

Visual and acoustic monitoring from static and on tow jack-up drilling rigs and production platforms was carried out over 55 non-consecutive days between 2004 and 2014.

### Weather conditions

Forecast weather and empirical North Sea buoy data were correlated positively (Table 7). Thus, forecast weather was considered an accurate representation of weather conditions during all monitoring periods.

**Table 7:** Results of empirical buoy weather and forecast weather comparisons.

Tow	Variable	Test	CC	P value	No. of samples
T2	Significant wave height	S	0.892	< .001	30
T3	Significant wave height	P	0.946	< .001	12
T3	Wind speed	P	0.963	< .001	12
T4	Wind speed	S	0.622	< .001	24
T5	Wind speed	S	0.836	< .001	24
S3	Significant wave height	S	0.816	< .001	40

T2 = tow two from L3 to L4 in 2009/2010; T3 = tow three from L5 to A6-A in 2012; T4 = tow four from Wingate to A6-A in 2014; T5 = tow five from A6-A to Wingate in 2014. S3 = L4 in 2010; S = Spearman’s Rank Correlation Coefficient; P = Pearson’s correlation; CC = Correlation Coefficient.

**Fig 2:**
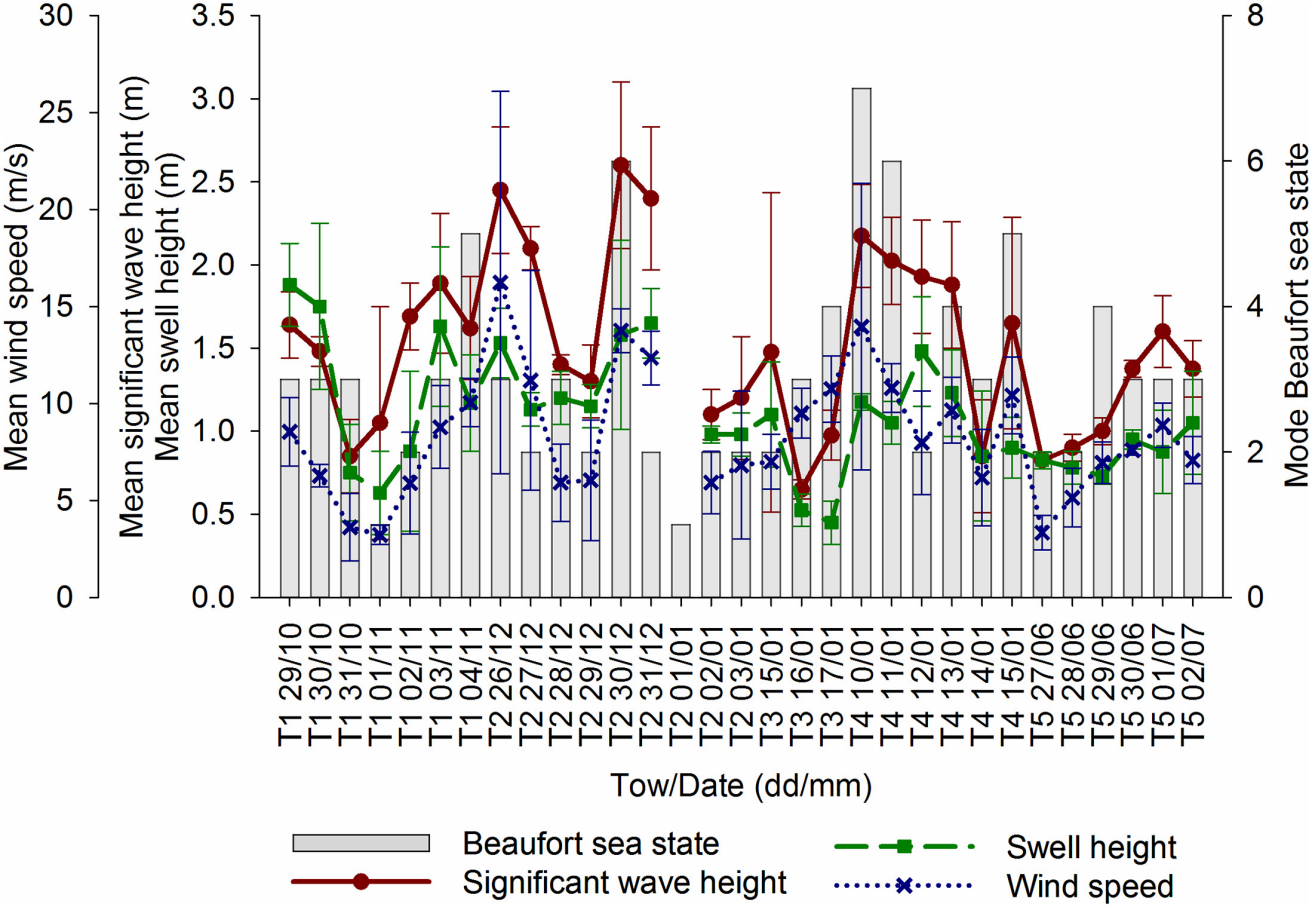
Mean ± standard deviation for weather conditions during tows. T1 = tow one from L1 to L2 in 2004; T2 = tow two from L3 to L4 in 2009/2010; T3 = tow three from L5 to A6-A in 2012; T4 = tow four from Wingate to A6-A in 2014; T5 = tow five from A6-A to Wingate in 2014. Graphs produced in Sigmaplot [48].

**Fig 3:**
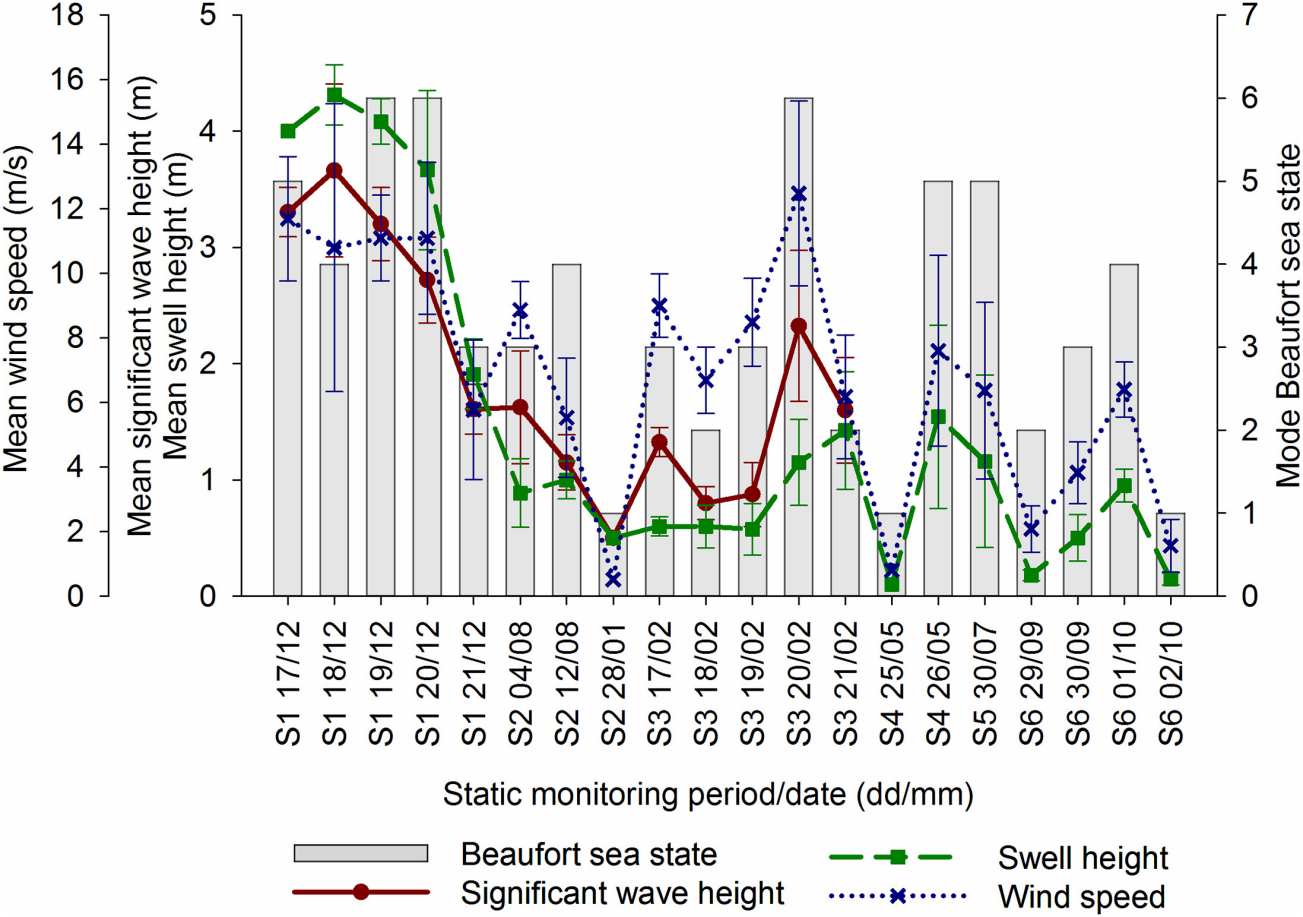
Mean ± standard deviation for weather conditions during static monitoring periods. S1 = L2 in 2004; S2 = A6-A August 2005 & January 2006; S3 = L4 in 2010; S4 = L6 in 2013; S5 = L7 in 2013; S6 = L8 in 2014. Graphs produced in Sigmaplot [48].

**Fig 4:**
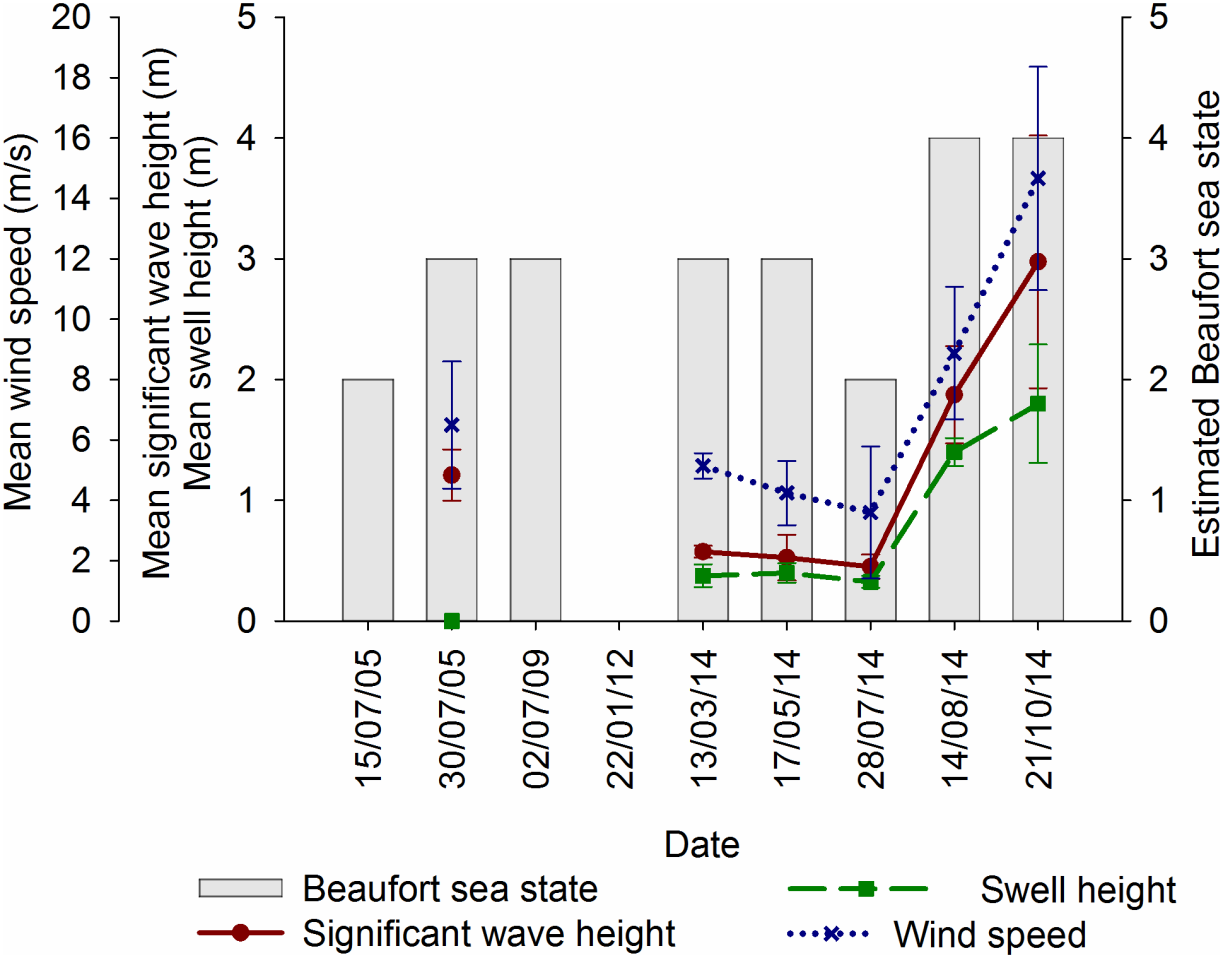
Mean ± standard deviation for weather conditions for days with incidental sightings. Graphs produced in Sigmaplot [48].

Weather conditions varied throughout all monitoring periods; forecast significant wave height, swell height, wind speed, and empirical on-site Beaufort sea state are displayed graphically for tows in Fig 2, static monitoring periods in Fig 3, and for incidental sightings in Fig 4.

In total, 38% (12 days) of visual monitoring during tows was conducted in conditions considered conducive for visual monitoring of marine mammals (mode Beaufort sea state <2, and swell heights <2 m). For static monitoring periods, 30% (6 days) of visual monitoring was also carried out in good conditions, and 30% during incidental sightings.

**Fig 5:**
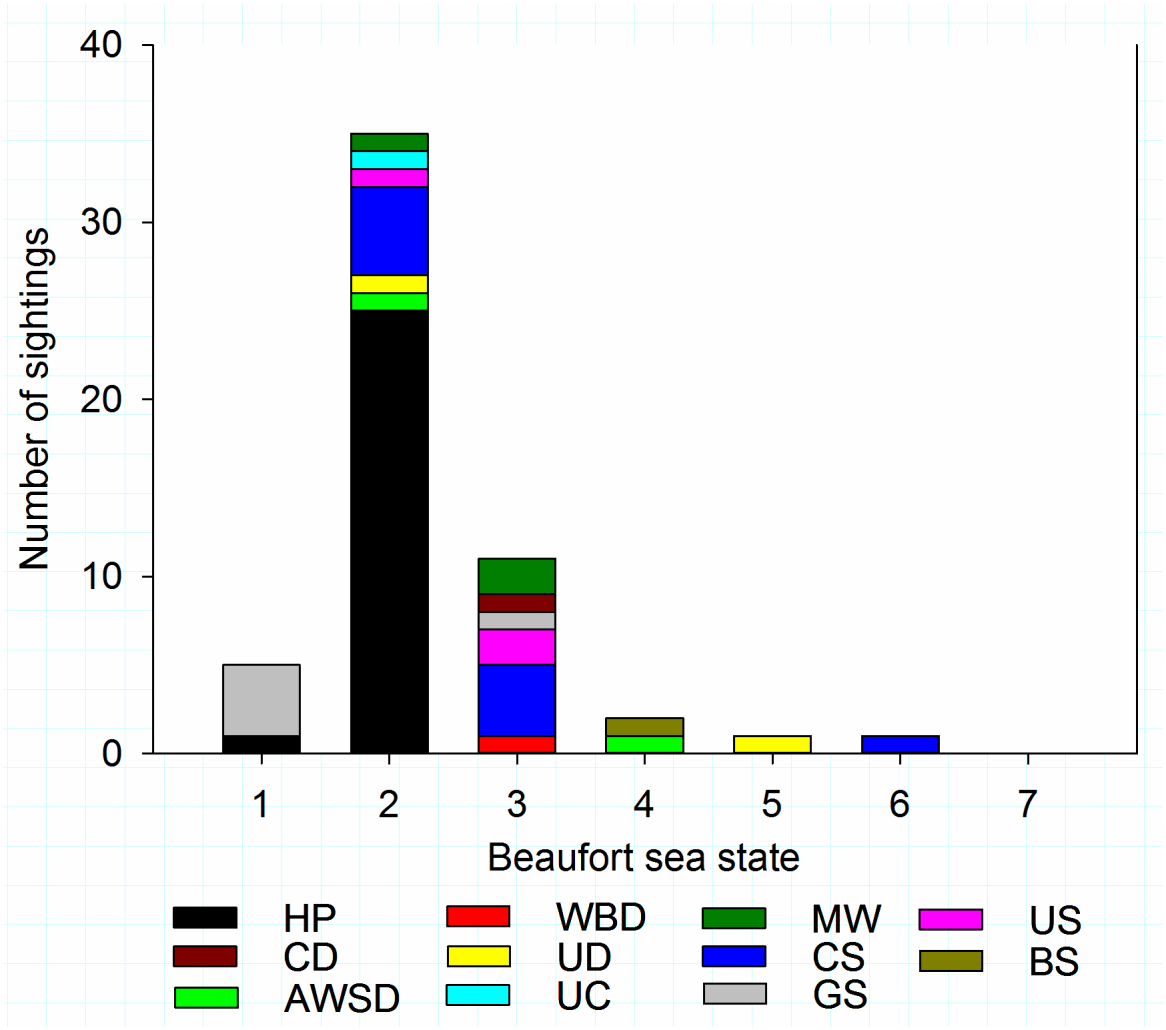
A breakdown of marine fauna sightings per Beaufort sea state. HP = harbour porpoise; CD = common dolphin (*Delphinus delphis*); AWSD = Atlantic white sided dolphin (*Lagenorhynchus acutus*); WBD = white beaked dolphin (*Lagenorhynchus albirostris*); UD = unidentified dolphin; UC = unidentified cetacean; MW = minke whale (*Balaenoptera acutorostrata*); CS = common seal (*Phoca vitulina*); GS = grey seal; US = unidentified seal; BS = basking shark (*Cetorhinus maximus*). Graphs produced in Sigmaplot [48].

Number of sightings was correlated negatively with Beaufort sea state (Spearmans rank correlation coefficient CC = -0.883, P = 0.000000200, n = 7). A total of 73% of all sightings (species combined) occurred in Beaufort sea states <2 and 93% in Beaufort sea state <3. All harbour porpoise sightings occurred in Beaufort <2, but dolphins were sighted in sea states up to a Beaufort 5, and seals up to Beaufort 6. A breakdown of sightings per Beaufort sea state is displayed in Fig 5.

### Marine Mammal Observation

Approximately 238 hours of visual observations were carried out by MMOs on 45 days, spanning five tows and five static monitoring periods. A total of 47 marine mammal sightings were recorded by MMOs on dedicated watch. Ten incidental sightings of marine megafauna were reported over 10 years.

#### On tow

**Fig 6:**
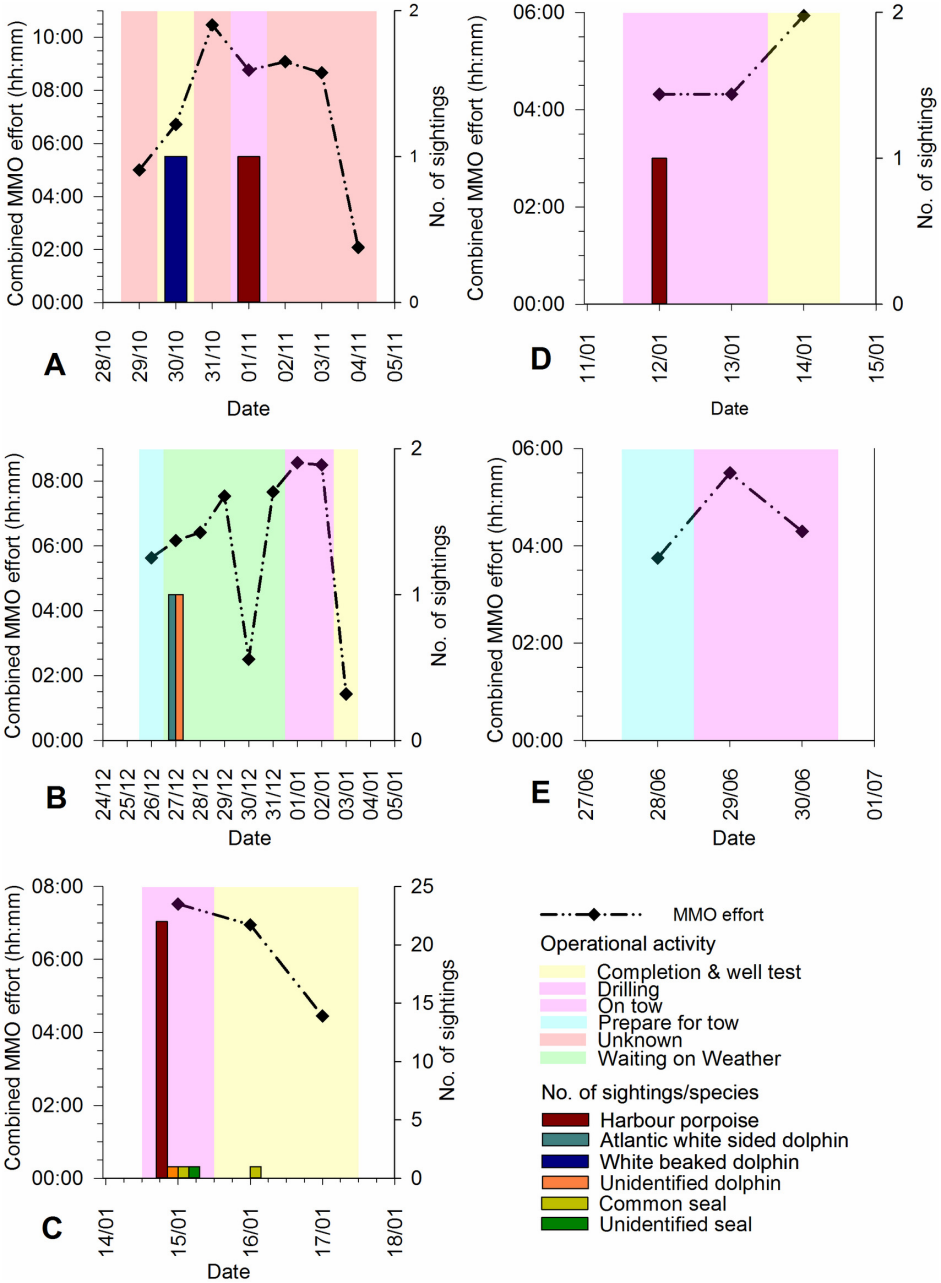
Marine Mammal Observer (MMO) effort, visual sightings, and operational activity, during tows one to five. T1 = tow one from L1 to L2 in 2004; T2 = tow two from L3 to L4 in 2009/2010; T3 = tow three from L5 to A6-A in 2012; T4 = tow four from Wingate to A6-A in 2014; T5 = tow five from A6-A to Wingate in 2014. Graphs produced in Sigmaplot [48].

**Fig 7:**
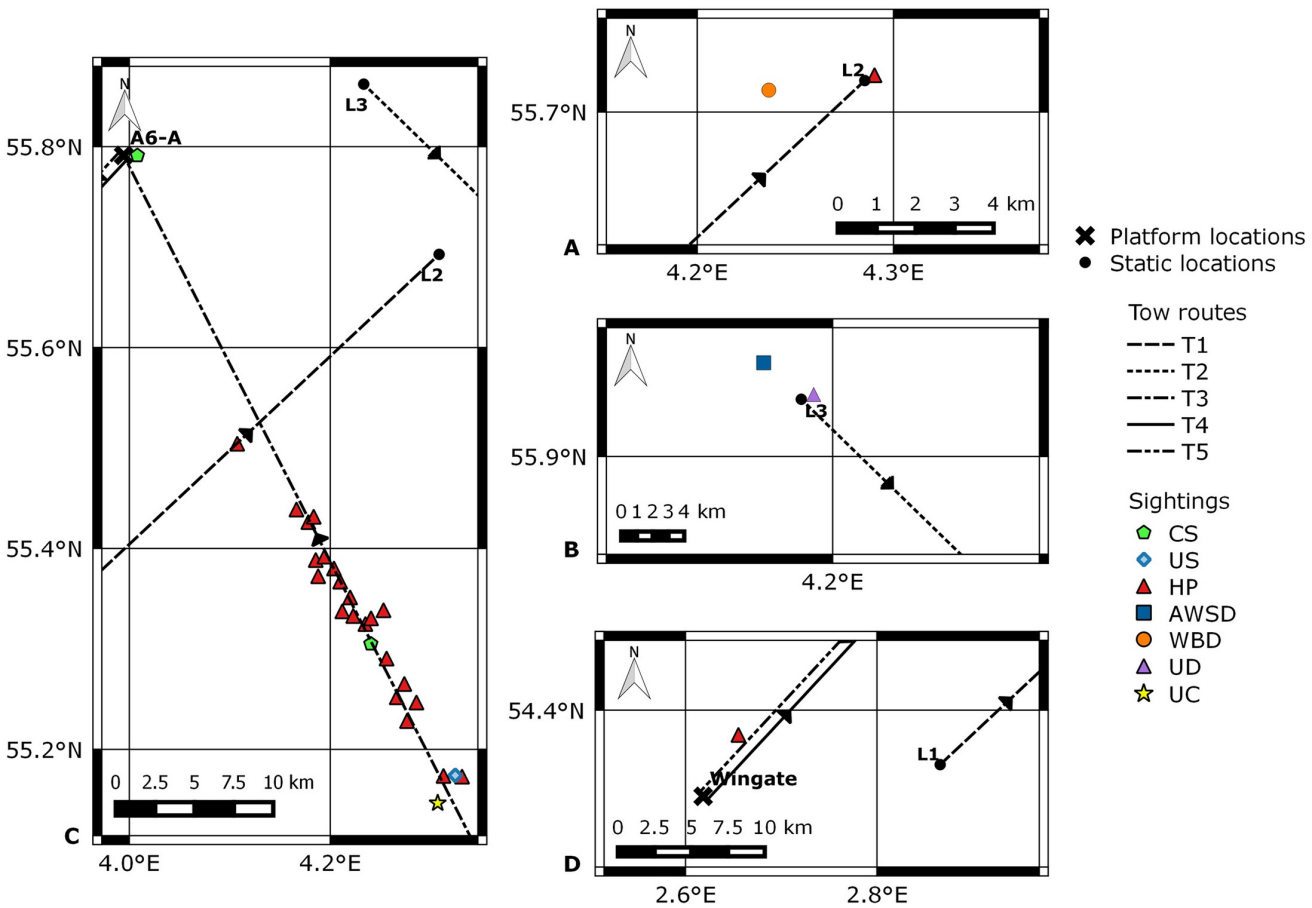
Locations of visual sightings recorded whilst drilling rigs were on tow. T1 = tow one from L1 to L2 in 2004; T2 = tow two from L3 to L4 in 2009/2010; T3 = tow three from L5 to the A6-A in 2012; T4 = tow four from Wingate to A6-A in 2014. CS = common seal; US = unidentified seal; HP = harbour porpoise; AWSD = Atlantic white sided dolphin; WBD = white-beaked dolphin; UD = unidentified dolphin; UC = unidentified cetacean. Chart produced in QGIS [31].

Marine Mammal Observers conducted visual observations during daylight hours on 25 days, during five tows (T1–T5) between 2004 and 2014 (Fig 1). Marine Mammal Observer effort, sightings, and operational activity for all tows are displayed in Fig 6, and sighting locations in Fig 7.

There were 32 sightings of 59 animals in total. Species included 24 sightings of 46 harbour porpoises, one pod of four white beaked dolphins, one sighting of two Atlantic white sided dolphins, and three common seals. One unidentified dolphin was recorded during T2, and an unidentified cetacean and unidentified seal were seen during T3.

Sightings occurred before, during, and after rig tows. Two sightings, both of dolphins in 2009, occurred prior to T2 (Fig 6B), whilst the NGS was Waiting on Weather (WoW) and conducting general maintenance activities.

The majority of sightings (26) occurred whilst rigs were on tow, with 25 sightings recorded in one day in 2012 (15/01/12), 22 of which were harbour porpoises (Fig 6C). One sighting of a harbour porpoise also occurred 1,500 m from the NGS during T4 (Fig 6D).

Four sightings were recorded after rig tows when the jack-up drilling rig was static; two in 2004 (Fig 6A) and two in 2012 (Fig 6C). In 2004, four white beaked dolphins were seen from the NK whilst it was undergoing pre-loading activities, and a harbour porpoise was sighted once drilling had resumed. In 2012, a common seal was seen from the NGS; the rig was jacking at the time.

#### Static

**Fig 8:**
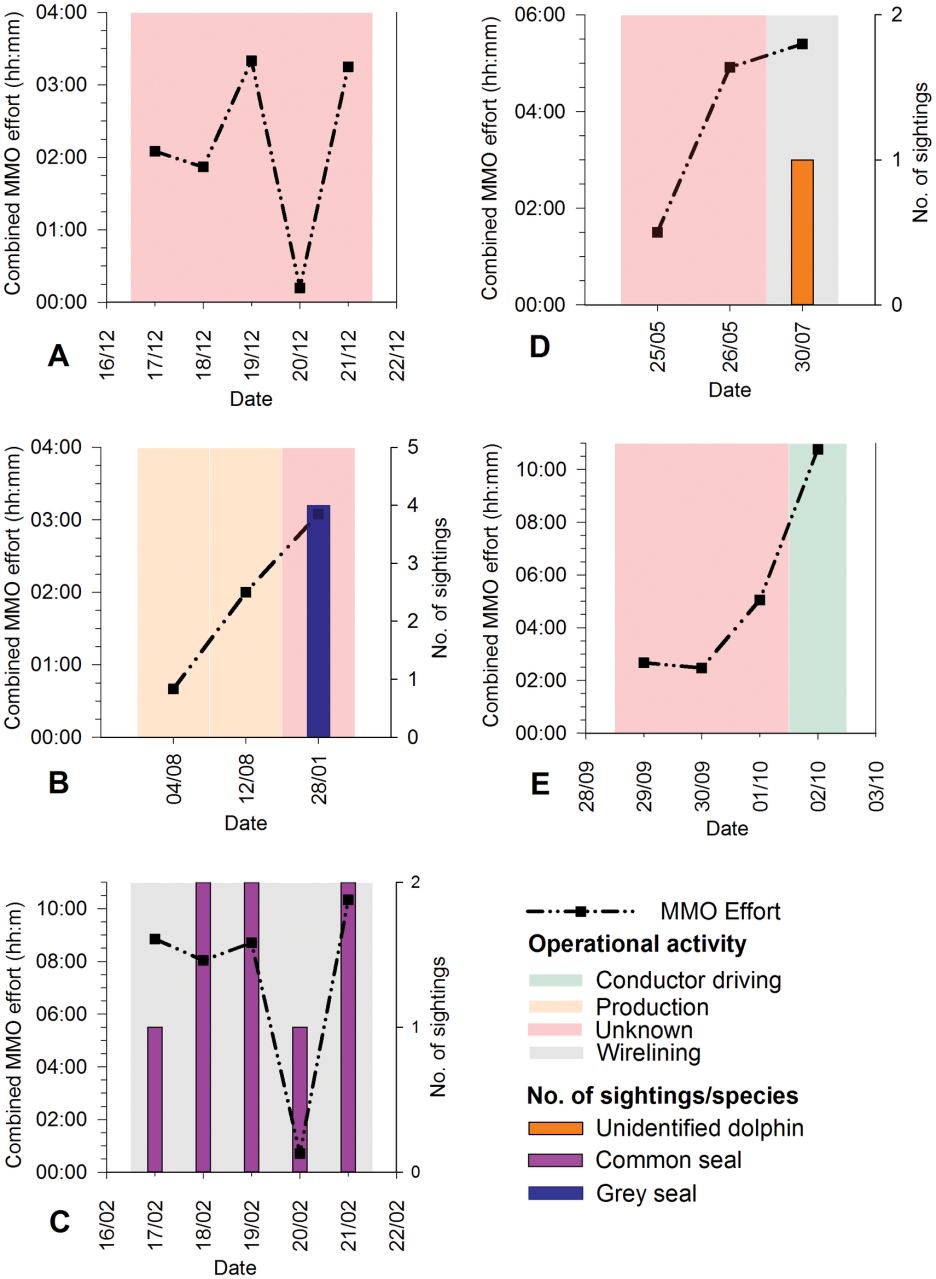
Marine Mammal Observer (MMO) effort, sightings and operational activity for static monitoring periods. A = S1 (L2 in 2004); B = S2 (A6-A August 2005 & January 2006); C = S3 (L4 in 2010); D = S4 (L6 in 2013 & S5 (L7 in 2013)); E = S6 (L8 in 2014). Graphs produced in Sigmaplot [49].

**Fig 9:**
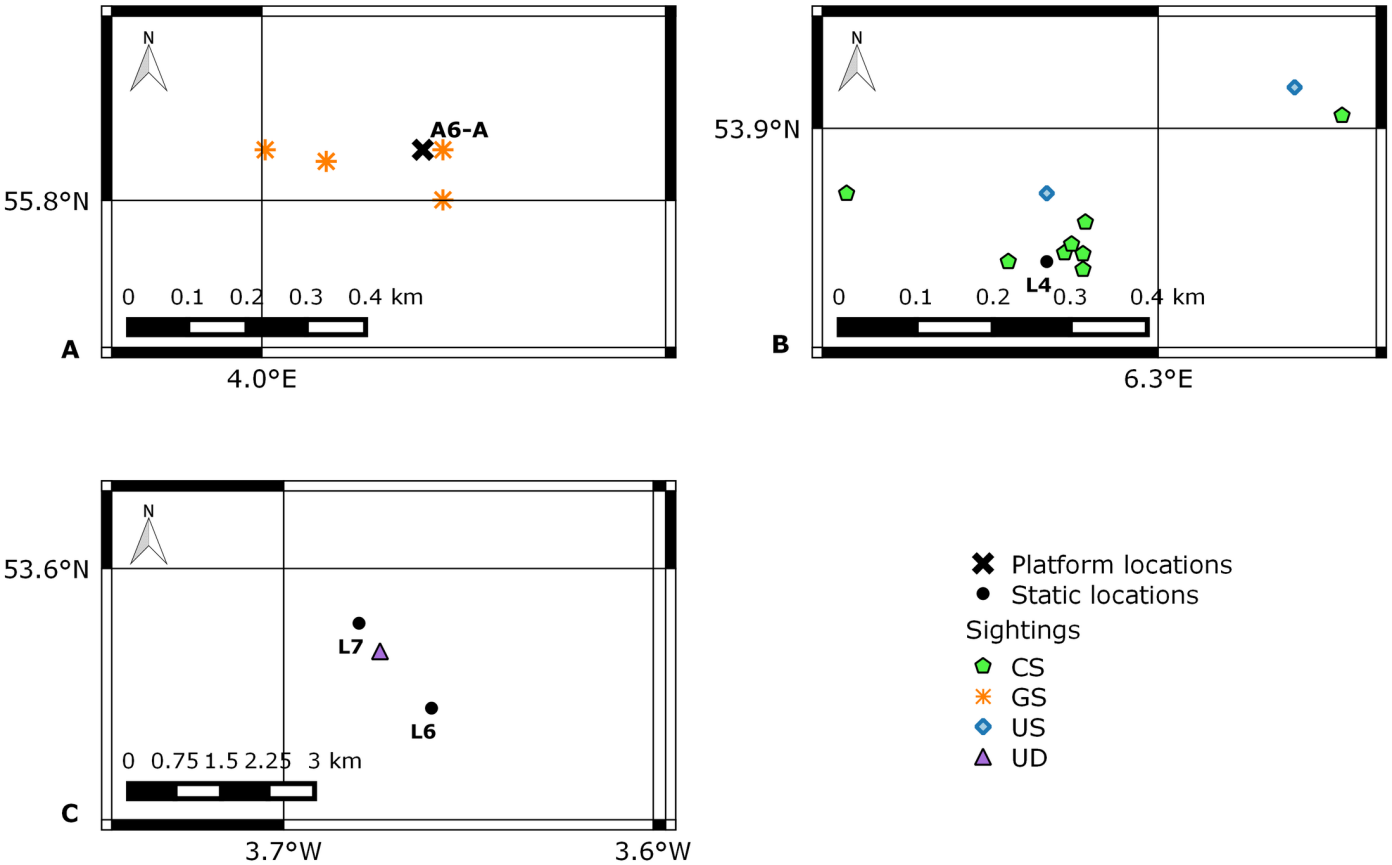
Location of sightings from static installations. CS = common seal; GS = grey seal; US = unidentified seal; UD = unidentified dolphin. Chart produced in QGIS [31].

Marine Mammal Observers conducted visual observations from static jack-up drilling rigs and production platforms during daylight hours on 20 days, across six monitoring periods (S1–S6) between 2004 and 2014. Marine Mammal Observer effort, sightings, and operational activity for all static monitoring periods are displayed in Fig 8, and locations of sightings in Fig 9.

There were 15 marine mammal sightings of 15 animals seen by MMOs whilst on duty (dedicated watch). Species included four grey seals (*Halichoerus grypus*), two unidentified seals, eight common seals, and an unidentified dolphin.

Operational activity at the time of sightings was either wirelining or production, with one sighting during an unknown activity.

#### Incidental

**Fig 10:**
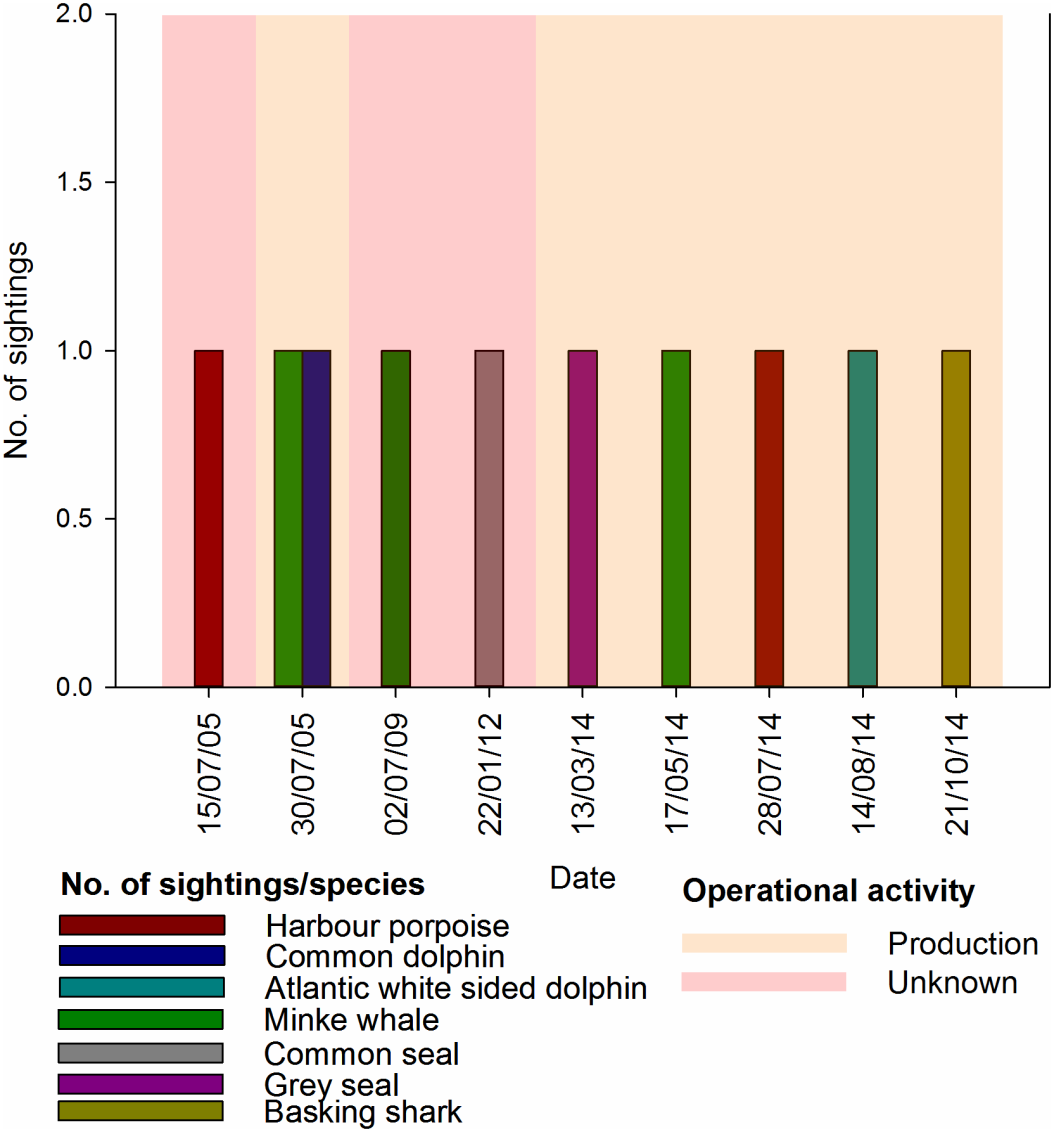
Incidental sightings and operational activity. Graphs produced in Sigmaplot [48].

**Fig 11:**
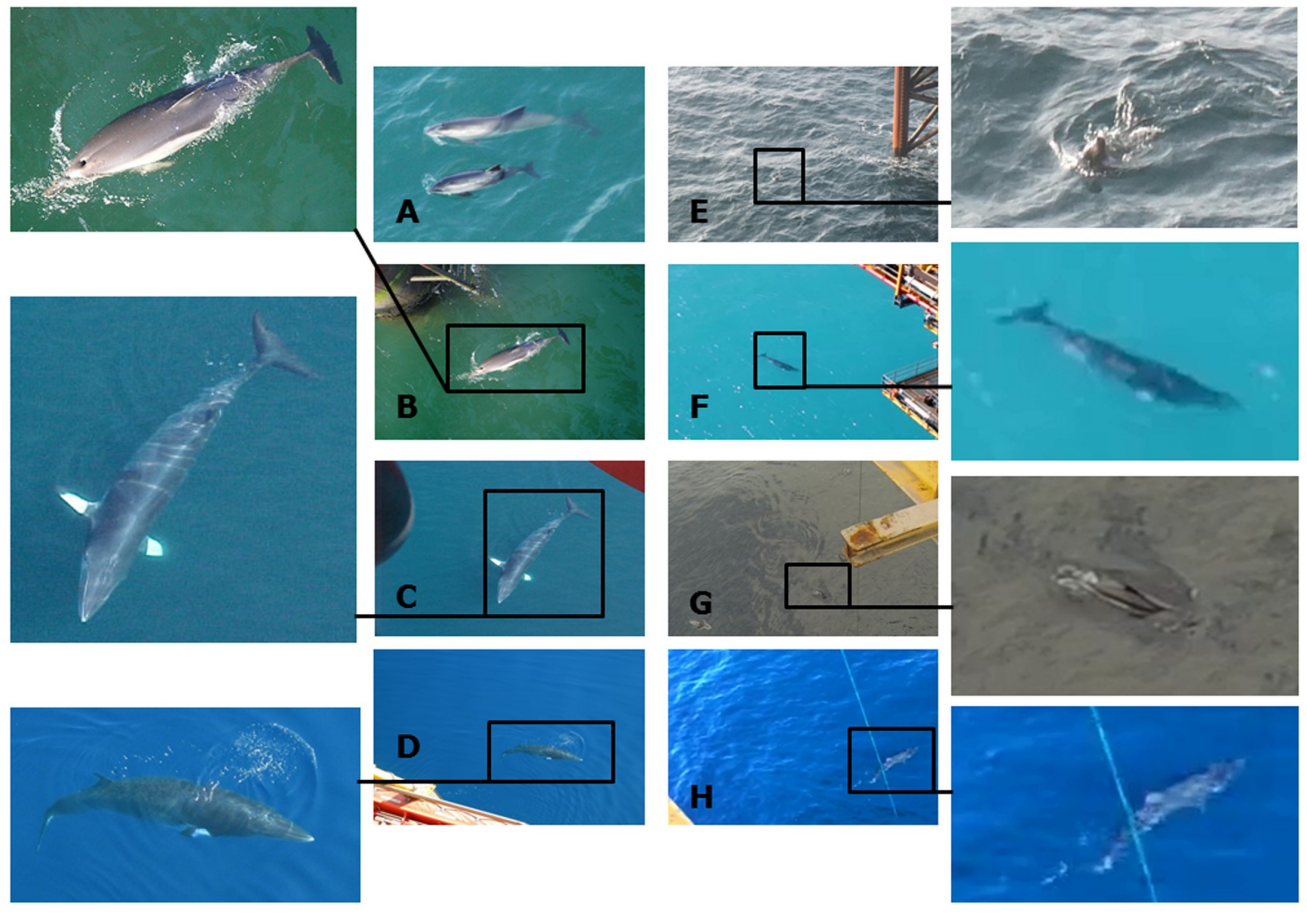
Photographic evidence of incidental visual sightings recorded from the A6-A production platform. A = mother and calf harbour porpoise in 2005; B = common dolphin in 2005; C = minke whale in 2005; D = minke whale in 2009; E = grey seal in 2014; F = minke whale in 2014; G = super pod of Atlantic white sided dolphins in 2014; H = basking shark in 2014.

Ten incidental marine fauna sightings of 109+ animals were recorded from the A6-A over the ten years (Fig 10). Species included one common seal, one grey seal (Fig 11E), two sightings of three harbour porpoises (including one calf) (Fig 11A), a common dolphin (Fig 11B), a super pod of Atlantic white-sided dolphins (Fig 11G), three sightings of single minke whales (Fig 11C, 11D and 11F), and a basking shark (Fig 11H).

Limited auxiliary data were recorded for incidental sightings, but photographic evidence was collected of eight of the ten sightings (Fig 11). All animals were within the 500 m exclusion zone, and many approached within a few metres and/or passed between the installations’ legs.

### Passive Acoustic Monitoring

**Fig 12:**
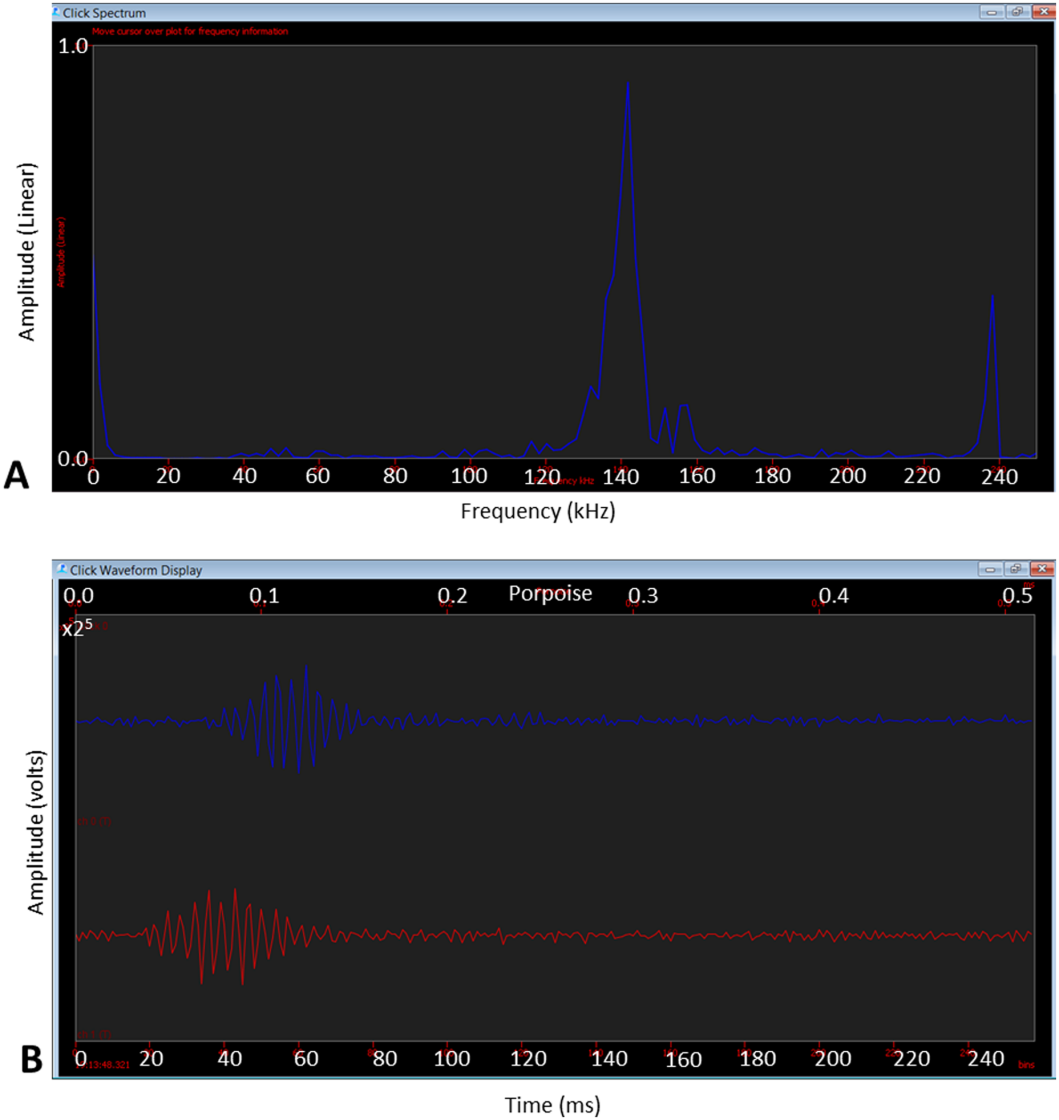
A harbour porpoise click spectrum (A) and wave form (B) as viewed in PAMGuard.

Real-time towed PAM was conducted in 2014 only. The hydrophone array was deployed for approximately 160 hours over 12 days. A total of 308 individual clicks were identified as harbour porpoises; an observed example of a typical waveform and spectrogram from the data is displayed in Fig 12.

**Fig 13:**
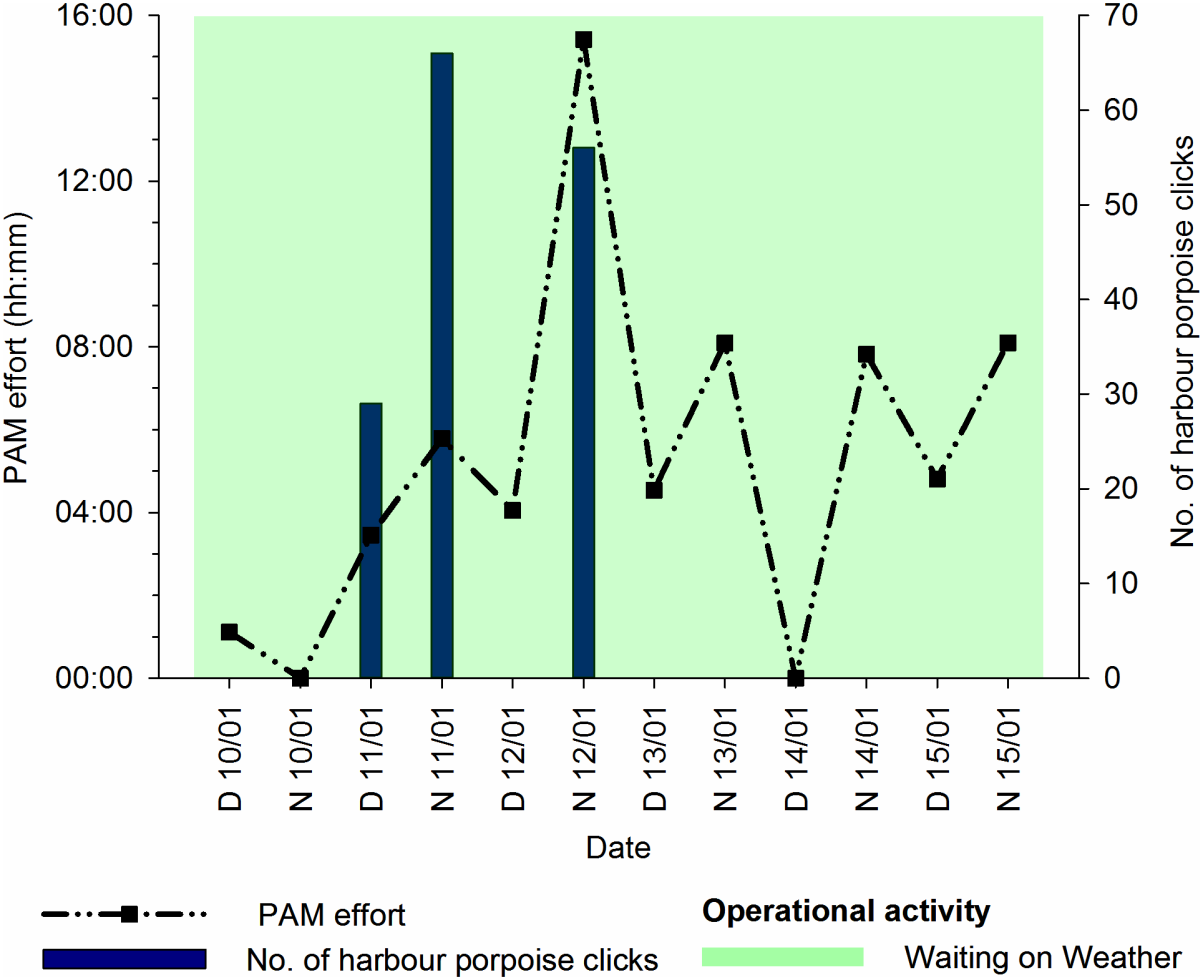
Passive Acoustic Monitoring (PAM) effort, number of harbour porpoise clicks, and operational activity for T4. T4 = Tow 4 from Wingate to A6-A in January 2014; D = day; N = night. Graphs produced in Sigmaplot [48].

In January, approximately 63 hours of recordings were made. A summary of effort, detections, and operational activity is displayed in Fig 13. A total of 151 harbour porpoise clicks were recorded, which equated to 14 click events or 30 Porpoise Positive Minutes (PPM). All detections occurred prior to T4, whilst the NGS was stationary, WoW, and undergoing general rig maintenance. The majority (80%) of clicks were recorded overnight, when visual observations were not possible, but two single clicks and one click event (27 clicks) occurred during daylight hours (approximately 08:05–15:50 UTC); however, sea state was Beaufort 5, and MMOs were off-effort, so porpoises were not sighted visually.

Inter Click Intervals (ICI) varied considerably. The Minimum Inter Click Intervals (MICI) of click events varied between 0.013–0.54 s, which is not indicative of inferred feeding behaviour.

**Fig 14:**
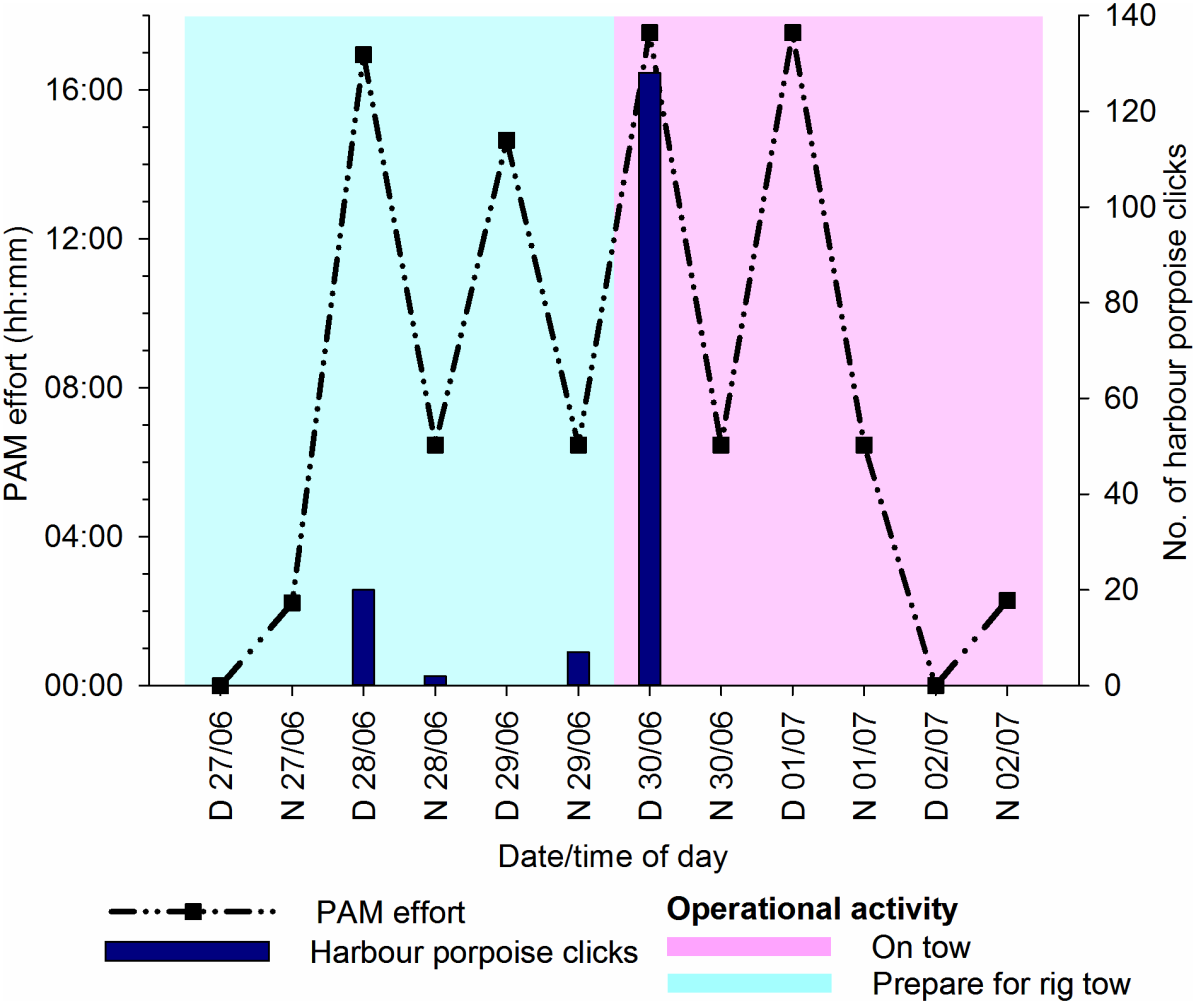
Passive Acoustic Monitoring (PAM) effort, number of harbour porpoise clicks, and operational activity for T5. T5 = tow five from A6-A to Wingate, in June 2014; D = day; N = night. Graphs produced in Sigmaplot [48].

**Fig 15:**
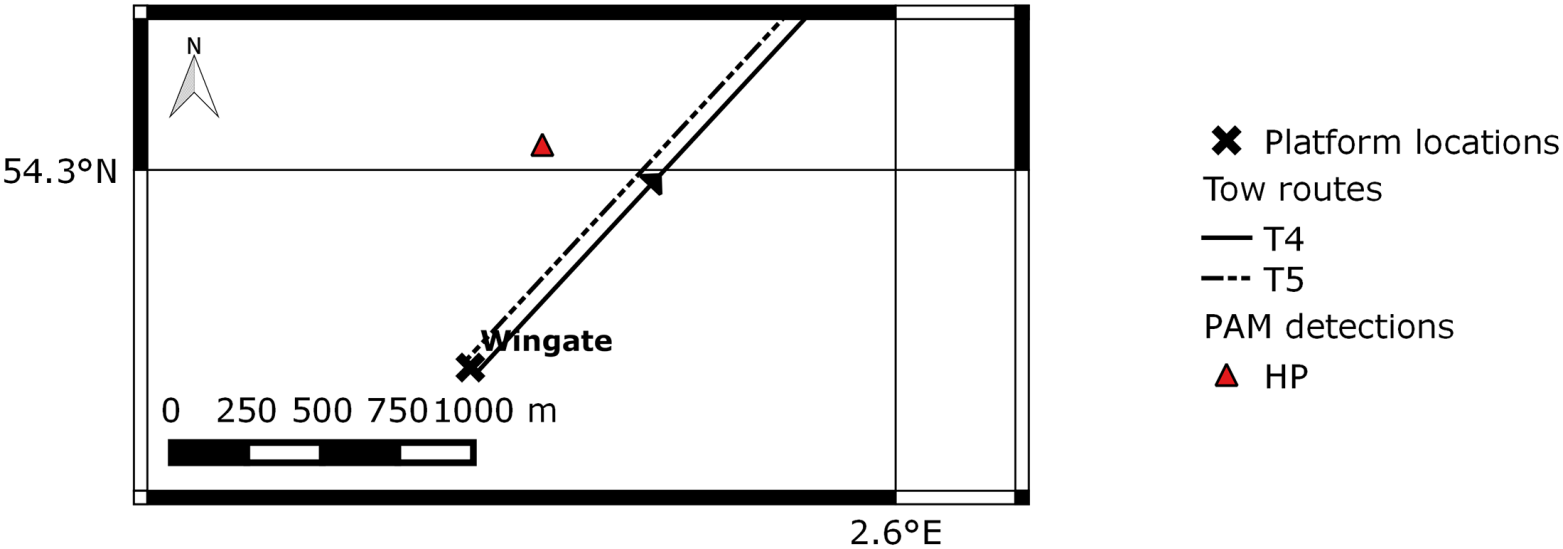
Projected location of the harbour porpoise click event recorded during T5. Arrow denotes direction of movement. T4 = tow four from Wingate to A6-A in 2014; T5 = tow five from A6-A to Wingate in 2014; HP = harbour porpoise. Map produced in QGIS [31].

In June, approximately 97 hours or recordings were made. Effort, detections, and operational activity are summarised in Fig 14. A total of 157 individual harbour porpoise clicks were recorded, which equated to 7 click events or 20 PPM. Of the 157 clicks, 29 were recorded whilst the NGS was stationary and located at the A6-A, prior to T5, but the remaining 128 clicks (9 PPM) were recorded whilst the rig was on tow. Clicks occurred towards the end of the tow, when the NGS was approximately 1.2 km from the Wingate platform, but prior to any joining activity. Start and end location of on-tow detections are displayed in Fig 15.

Similar to January, ICIs were varied, with MICIs of 0.009–0.73 s observed within click events. There were two click events with MICIs of 9.06 ms on 28/06/14.

## Discussion

Marine mammals are likely recorded on a regular basis by MMOs and PAM Operators during routine noise mitigation procedures around jack-up drilling rigs and production platforms, but data are restricted to confidential client reports. There is only one peer-reviewed marine mammal study (also from the A6-A/NK) in the North Sea [24], no peer-reviewed marine mammal reports from offshore installations in the Irish Sea, and one peer-reviewed marine mammal study from around offshore installations in the Adriatic [25], the latter also reporting potential elevated dolphin feeding behaviour. Visual and acoustic detections reported within this study therefore provide important additional evidence that marine mammals are present around offshore installations, and appear to be the first such acoustic detections obtained from a North Sea drilling rig and rig-platform complexes whilst using a typically configured hydrophone array designed for towing in combination with real-time PAMGuard software.

The decade of visual monitoring data, and acoustic data from 2014 are typical of industrial data, as the primary focus of MMOs and PAM Operators is mitigation for industrial activities [45], rather than dedicated research. Therefore, monitoring periods are sporadic and of limited duration (1–9 days). Nevertheless, these brief opportunities are a valuable, hitherto unused resource, which provides further evidence that marine megafauna are present around O&G installations.

Past and current long-term T-POD and C-POD datasets [24], indicate porpoise acoustic presence around North Sea installations can be high. Reasons for low visual sightings in this study are likely twofold. Firstly, sea state affects sighting probability substantially. Weather offshore is optimal rarely, and daily mode Beaufort sea states during these observations were ≥3 on 62% of days. Below Beaufort sea state 2, likelihood of marine mammal sightings is higher, regardless of species; however, higher sea states reduce sighting probabilities rapidly for all species, especially the harbour porpoise [50–52]. Consequently, in this study, no porpoises were sighted in Beaufort sea state >2, but dolphins were sighted up to Beaufort 5, and seals in Beaufort 6. Secondly, [24] show that harbour porpoises are more active acoustically around installations at night. Visual MMO observations are only carried out in daylight, when acoustic detections of porpoises around installations has been shown to be lower compared to during the night [24]. The 2014 data set is small, but porpoises were detected acoustically overnight by PAM Operators, which augments prior observations at the same platform, A6-A. Analysis of T-POD and C-POD data sets collected simultaneously with real-time PAM again at the A6-A are currently being undertaken.

It is not standard procedure for marine crew, who are focussed on their own professional responsibilities, to report incidental sightings of marine megafauna sightings when MMOs are not aboard installations; consequently, it is highly conceivable that more sightings occurred than were reported, corroborated frequently during verbal discussions with marine crew, who do not record dates, times, visual descriptions. These are the first confirmed sightings of all species from offshore installations in the North Sea.

One visual sighting of a harbour porpoise occurred at a distance of 1,500 m from the NGS, whilst PAM was recording continuously. The harbour porpoise emits Narrow Band High Frequency (NBHF) echolocation clicks [27–29], which attenuate rapidly; more specifically, on account of sound propagation physics (not technology limitations), the harbour porpoise needs to be within 250–300 m of the hydrophone (and on-axis) to be detected, so this particular animal was beyond possible detection range of PAM. Limitations of PAM in an industrial context are discussed in [45].

The harbour porpoise was the only species detected acoustically by PAM, whilst being monitored in real-time by PAM Operators and no other species were sighted by MMOs whilst the PAM equipment was deployed. Low frequency data were recorded, however not reviewed post deployment, so no inferences are made about acoustic detections of other species. During tows, hydrophone depth varied considerably, regularly akin to a whipping motion that spanned the entire water column of approximately 30 m (in only a matter of seconds), as the non-hydrodynamic nature of rig barges created turbulence; consequent noise masking of biological signals almost certainly contributed to reduced numbers of detections.

The reef effect of rigs and platforms, coupled with the 500 m fishing exclusion zone, renders installations potential foraging habitats for marine megafauna. Presence of porpoise feeding buzzes in T-POD and C-POD datasets reported by Todd, Pearse [24], have shown that harbour porpoises are potentially feeding around the legs of platforms in the North Sea. Data reported here are not sufficient to conclude that marine mammals were feeding; however, porpoises are small, with limited body fat, and relatively high metabolic rates compared to other cetaceans. Food consumption in porpoises varies between 4–9.5% [53] and 7.5–10% body weight daily [54], representing between 8,000 and 25,000 kJ/day [53]. The small size of porpoises does not enable them to carry large energy stores [55], so their patterns of movement are likely related strongly to distribution of their prey. Maintaining high feeding rates is particularly important for lactating females. It is conceivable, therefore, that the mother and calf sighted in close proximity to the A6-A (located approximately 250 km from land), were targeting the platform as a temporally-spatially predictable, dependable, and potentially plentiful food source, which could provide the mother with a reliable milk source for her calf.

Likewise, whilst it cannot be concluded from the data that other marine megafauna were foraging, whale sharks (*Rhincodon typus*) have been observed feeding on surface zooplankton around an offshore installation located within the Al Shaheen oil field off the coast of Qatar [56], and as such it is conceivable that the basking shark observed in this study was doing the same.

There is a possibility that porpoises detected visually and acoustically towards the beginning/end of a tow were following jack-up rigs as mobile feeding stations; however, without individual recognition, perhaps in the form of mark-recapture-porpoise-satellite-tagging experiments, this hypothesis cannot be tested. It is entirely feasible, given known sustained swimming speeds of porpoises, approximately 0.7–2.2 m sec^-1^ [57, 58] and typical rig tow speeds of 4–5 kts (2–3 m sec^-1^) that animals could keep up with rigs on-tow. Indeed, as yet unpublished T-POD and C-POD data from the NK and NGS record animals immediately after jacking down, indicating animals are at the location straight away. Further research would need to be undertaken on potential porpoise prey swimming speeds, or known behaviour of rig-aggregated fish species following rig departure from a typical 2–3 month drilling campaign. It is also possible that once a rig has departed a location, porpoise-fish-prey species, particularly those less mobile, such as gobies (Gobiidae) and small gadoids (cod-like species) deprived of rig refugia, may be more easily accessible targets for any porpoises remaining in the vicinity. Presence of marine mammals on tows may also be related to wider local oceanographic conditions, not examined here.

Marine fauna observed visually or acoustically from the stationary platforms, whilst rigs were attached to platforms pre rig-tow, or on rig approach to platforms, were possibly already in the vicinity, potentially maximising prey availability from a ‘reef’ effect from established platforms in situ for relatively longer periods, but that cannot be concluded from data presented here.

Visual and acoustic detections presented here provide further evidence that O&G installations are frequented by marine megafauna. Further research is required to determine importance of such areas, but presence alone has implications for decommissioning. Prior to removal, a decommissioning programme, discussed in detail within DECC [59], must be produced and approved under The Petroleum Act 1998; one such aspect is the production of an Environmental Impact Assessment (EIA), and aside from potential impacts of explosions on marine mammals, megafauna have, for the most part, been overlooked in this process. If marine megafauna are utilising installations as foraging areas, as demonstrated by Todd, Pearse [24] for harbour porpoises in the North Sea, or by Robinson, Jaidah [56] for whale sharks in Qatar, full removal is likely removing reliable foraging habitats. Consequently, visual and acoustic data collected from installations themselves should be utilised during the production of EIAs to better assess potential long-term impacts of decommissioning.

## Supporting Information

S1 File Acknowledgement for use of EMODNet data in publications. (PDF)
