# Supplementary material for: Meals on Wheels? A Decade of Megafaunal Visual and Acoustic Observations from Offshore Oil & Gas Rigs and Platforms in the North and Irish Seas
Source: PLoS One. 2016 Apr 14;11(4):e0153320. doi: 10.1371/journal.pone.0153320 (PMC4831756; doi:10.1371/journal.pone.0153320)
Supplement: S1 File — (PDF) [file pone.0153320.s001.pdf]

Acknowledgement for use of EMODNet data in publications.

The bathymetric metadata and Digital Terrain Model data products have been derived from the EMODnet Bathymetry portal - http://www.emodnet-bathymetry.eu.

This portal was initiated by the European Commission as part of developing the **European Marine Observation and Data Network** (EMODNet). The overall objective of EMODnet is to create pilots to migrate fragmented and inaccessible marine data into interoperable, continuous and publicly available data streams for complete maritime basins. The Bathymetry portal development started in June 2009 and now provides a range of options for freely browsing and downloading a harmonised Digital Terrain Model (DTM) for all European sea regions. The downloadable tiles are freely available in a number of formats. The EMODnet digital bathymetry has been produced from bathymetric survey data and aggregated bathymetry data sets collated from public and private organizations. These are processed and quality controlled. A further refinement and expansion is underway, by gathering additional survey data sets and where possible, upgrading the DTM grid resolution, and will result in new releases in time. The portal also includes a metadata discovery service that gives clear information about the background survey data used for the DTMs, their access restrictions, originators and distributors.
